# The Preventive Effect of Specific Collagen Peptides against Dexamethasone-Induced Muscle Atrophy in Mice

**DOI:** 10.3390/molecules28041950

**Published:** 2023-02-18

**Authors:** Jieun Oh, Sang Hee Park, Dong Seon Kim, Wooram Choi, Jiwon Jang, Laily Rahmawati, Won Young Jang, Hyun Kyung Lim, Ji Yeon Hwang, Ga Rin Gu, Jeong-Ho Geum, Su-Young Choi, Ji Hye Kim, Jae Youl Cho

**Affiliations:** 1 Department of Integrative Biotechnology, Sungkyunkwan University, Suwon 16419, Republic of Korea; 2 Department of Biocosmetics, Sungkyunkwan University, Suwon 16419, Republic of Korea; 3 COSMAX NS, INC., Seongnam 13487, Republic of Korea; 4 COSMAX NBT, INC., Seongnam 13487, Republic of Korea

**Keywords:** muscle wasting, myostatin, Smad2, AKT, mTOR, IL-6, anti-inflammation

## Abstract

Muscle atrophy, also known as muscle wasting, is the thinning of muscle mass due to muscle disuse, aging, or diseases such as cancer or neurological problems. Muscle atrophy is closely related to the quality of life and has high morbidity and mortality. However, therapeutic options for muscle atrophy are limited, so studies to develop therapeutic agents for muscle loss are always required. For this study, we investigated how orally administered specific collagen peptides (CP) affect muscle atrophy and elucidated its molecular mechanism using an in vivo model. We treated mice with dexamethasone (DEX) to induce a muscular atrophy phenotype and then administered CP (0.25 and 0.5 g/kg) for four weeks. In a microcomputed tomography analysis, CP (0.5 g/kg) intake significantly increased the volume of calf muscles in mice with DEX-induced muscle atrophy. In addition, the administration of CP (0.25 and 0.5 g/kg) restored the weight of the gluteus maximus and the fiber cross-sectional area (CSA) of the pectoralis major and calf muscles, which were reduced by DEX. CP significantly inhibited the mRNA expression of myostatin and the phosphorylation of Smad2, but it did not affect TGF-β, BDNF, or FNDC5 gene expression. In addition, AKT/mTOR, a central pathway for muscle protein synthesis and related to myostatin signaling, was enhanced in the groups that were administered CP. Finally, CP decreased serum albumin levels and increased TNF-α gene expression. Collectively, our in vivo results demonstrate that CP can alleviate muscle wasting through a multitude of mechanisms. Therefore, we propose CP as a supplement or treatment to prevent muscle atrophy.

## 1. Introduction

Muscle atrophy, also known as muscle wasting, is the thinning of muscle mass and can be caused by muscle disuse, aging, and diseases such as cancer and neurological problems [1,2,3,4,5,6]. Muscle atrophy is closely related to quality of life, and sarcopenia in elderly people leads to high morbidity and mortality. However, although the causes and treatment of muscle atrophy have been studied during the past few decades, therapeutic options for muscle atrophy are still inadequate [7,8]. Therefore, research to develop a therapeutic agent to treat a decrease in skeletal muscle mass is always required.

Myostatin is a myokine primarily secreted by muscle, and it is a member of the TGF beta family, which is highly conserved in mammals [9]. Functionally, myostatin has been demonstrated to negatively regulate muscle growth in various animal and human studies. Myostatin knockout mice showed a “double-muscle” phenotype [9]. In addition, humans with loss-of-function mutations in the myostatin gene showed increased muscle mass compared to normal individuals [10]. Moreover, solid evidence indicates that elevated myostatin contributes to the development of muscle atrophy [11], so targeting myostatin is considered a promising strategy for muscle wasting treatment. In skeletal muscle, myostatin signaling begins by binding to the activin type II (ActRII) B receptor. The interaction of myostatin and ActRII causes the phosphorylation and nucleus translocation of Smad to modulate the expression of target genes related to myogenesis and muscle protein metabolism, such as myogenic regulatory factors (MRFs), atrogin-1, and muscle RING finger 1 (MuRF1) [12,13]. The MRFs, which include myogenin, MyoD, Myf5, and MRF4, are key transcriptional factors that regulate the development and regeneration of skeletal muscle cells during myogenesis [14,15].

Irisin has been reported to be involved in muscle atrophy. Irisin, a cleaved form of fibronectin type III domain-containing protein 5 (FNDC5), is increased after exercise in rodents [16] and humans [17]. Irisin causes skeletal muscle hypertrophy by activating muscle satellite cells and protein synthesis [18]. Meanwhile, irisin injection recovered the loss of muscle mass in a denervation-induced muscle wasting rodent model [18]. Serum irisin levels were lower in women with sarcopenia, and were found to be negatively correlated with the quadriceps muscle cross-sectional area/body weight, which is an indicator of muscle mass [19]. In addition, irisin exerts anti-atrophic activity in DEX-treated myotubes [20]. These reports indicate irisin might have preventive roles in muscle atrophy and be potential biomarker.

Brain-derived neurotrophic factor (BDNF) is a growth factor that belongs to the neurotrophin family and has been studied extensively in the nervous system [21]. Interestingly, BDNF and neurotrophin are expressed in skeletal muscles, and circulating BDNF levels are upregulated after acute exercise [22,23,24]. In addition, BNDF expression increases after muscle injury, and elevated BNDF induces skeletal muscle regeneration in response to muscle damage [21,25]. Interestingly, a recent study reported that loss of muscle-specific BDNF is effective in endurance performance and increases muscle mass and function in sarcopenia [26]. We, therefore, assume that inhibiting the BDNF level could also be an interesting approach for muscle atrophy.

Muscle mass is maintained by dynamic control of protein synthesis and breakdown, and imbalance in those processes can lead to muscle atrophy. In muscle protein synthesis, the mechanistic target of rapamycin (mTOR) is a key regulator of various mechanical stimuli [27,28]. Amino acid (especially leucine) is one of the mTOR signaling stimulators and is known to be more potent than other stimulatory molecules, including insulin [29,30]. It is generally known that amino acids facilitate Rheb-induced activation by regulating the subcellular localization of mTOR [31,32]. Meanwhile, MuRF1 and atrogin-1 are muscle-specific E3 ligases involved in muscle degradation, and their expression is known to be transcriptionally upregulated in atrophic skeletal muscle [33]. FOXO transcriptional factors can induce MuRF1 and atrogin-1 gene expression during muscle atrophy [34,35]. In addition, myogenin has been reported to promote the expression of MuRF1 and atrogin-1 in denervation-induced muscle atrophy [36,37].

Protein supplements are one effective strategy for attenuating muscle wasting. Several in vitro and in vivo studies highlight the importance of nutritional strategies to ameliorate glucocorticoid-induced muscle atrophy. Oral administration of leucine or β-hydroxy β-methylbutyrate (HMB), a leucine metabolite, significantly inhibited muscle loss in DEX-treated Sprague-Dawley rats [38]. Consistently, supplementation with leucine also inhibits AMPK signaling, a protein synthesis inhibitory factor in rat skeletal muscle [39]. In addition, oral administration of a branched-chain amino acid (BCAA) suppresses DEX-induced muscle atrophy by reducing atrogin-1 expression and autophagy [40]. The efficiency of supplementation of amino acids on protein synthesis is further increased when accompanied by exercise. A recent study also found that a combination of endurance exercise and BCAAs promotes muscle protein synthesis [41,42]. These reports strongly suggest that adequate nutrient supply may be a key strategy for limiting muscle wasting and restoring muscle mass. On the other hand, collagen, which has a low amount of BCAAs, has been considered to have relatively low biological activity. However, recent studies have consistently reported that collagen peptides (CP) can increase muscle mass. In a randomized controlled trial of sarcopenic men (mean age 72.2 years) and premenopausal women (ages 15–50 years), CP supplements for 12 weeks in combination with resistance training significantly improved fat-free mass and leg muscle strength [43,44]. In addition, CP supplements increased fat-free mass in a cohort of young men (mean age 24 years) receiving resistance exercise training [45]. Furthermore, in a randomized controlled study of recreationally active male participants, post-exercise CP intake activated the muscle protein anabolic pathway in the vastus lateralis muscle [46].

Therefore, in this study, we sought to demonstrate the effect of specific CPs on muscle atrophy and its molecular mechanisms in an in vivo model. For this, we delivered DEX, a type of glucocorticoid, in high doses to mice to induce skeletal muscle atrophy and administered specific CP in combination with treadmill exercise. Using this in vivo model, we investigated specific CP’s preventive effects on muscle atrophy.

## 2. Results

### 2.1. CP Administration Beneficially Affects Muscle Volume and Weight in Mice with DEX-Induced Muscle Wasting

**Figure 1:**
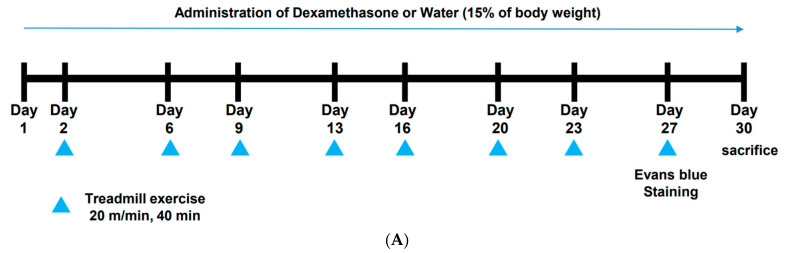

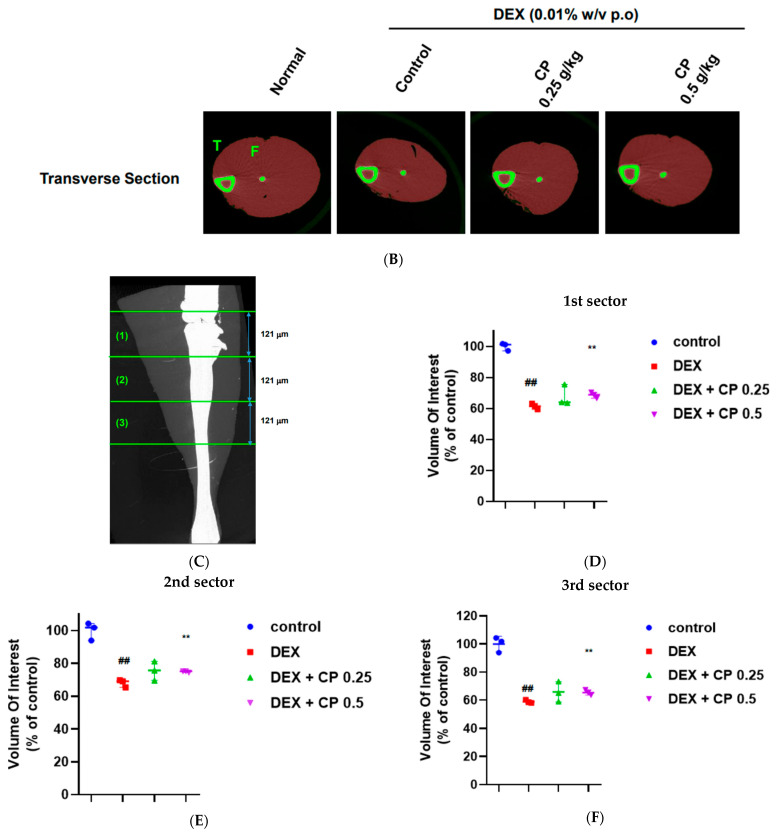

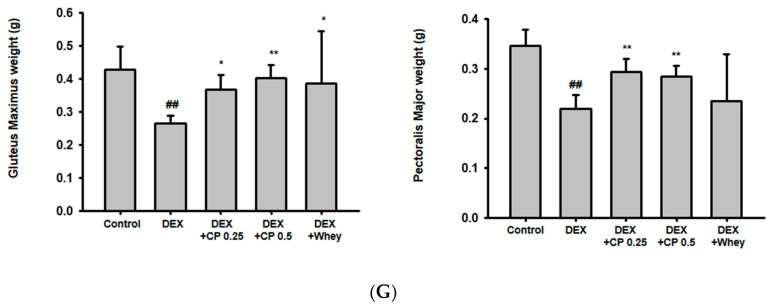
The beneficial effects of CP on muscle loss in mice with dexamethasone (DEX)-induced muscle wasting. (**A**) Experimental scheme to induce muscle atrophy and orally administer CP. (**B**) Micro-computed tomography (CT) images of calf muscles obtained after 4 weeks of CP intake. The muscles in red are shown as two-dimensional transverse section images. F and T represent the fibula and tibia, respectively. (**C**–**F**) The calf muscle volume in each region (**C**) was measured and presented in (**D**–**F**). (**G**) The weight of the gluteus maximus and pectoralis major, which were obtained and weighed after 4 weeks of CP intake. Whey protein (1 g/kg) was employed as a positive control. The data in (**D**–**G**) are expressed as the means ± SD. This experiment was performed with ten mice per group. ## *p* < 0.01, DEX vs. control. * *p* < 0.05, ** *p* < 0.01, DEX + CP (0.25 and 0.5) vs. DEX; CP, collagen peptides; DEX, dexamethasone; F, fibula; T, tibia.

To examine the effect of CP on muscle atrophy, we induced a phenotype similar to muscle atrophy in mice by administering DEX, and then we analyzed the degree of muscle loss using microCT. As shown in Figure 1B, calf muscle loss was observed in the DEX group, and orally administered CP (0.25 and 0.5 g/kg) decreased that muscle loss. We further measured the calf muscle volume to quantify the microCT images. After sectioning the calf muscle into three parts, we measured the volume of each region (Figure 1C) and found that 0.5 g/kg of CP significantly increased the muscle volume compared to that of the DEX group in all sections (Figure 1D–F). In addition, CP (0.25 and 0.5 g/kg) significantly increased the weight of the gluteus maximus and pectoralis muscles that were reduced by DEX, and the effect of CP was superior to that of whey protein (1 g/kg) (Figure 1G).

### 2.2. CP Intake Increases Fiber CSA in Mice with DEX-Induced Muscle Atrophy

**Figure 2:**
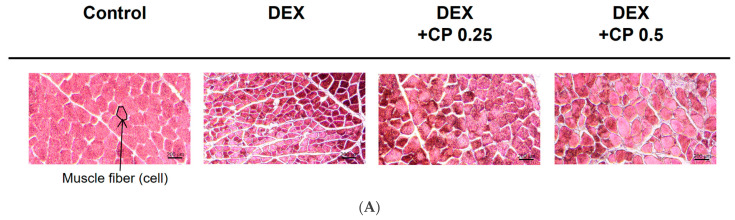

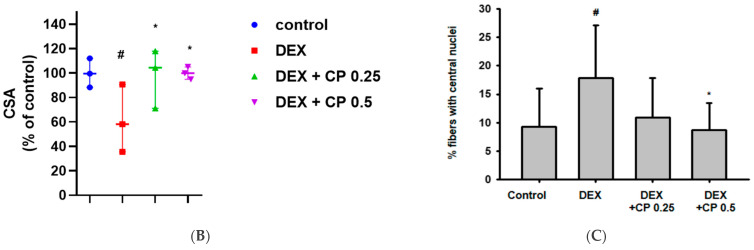
Effect of CP on histological features and cross-sectional area (CSA) of muscle fibers. (**A**) Representative H&E-stained images of calf muscles from each group. The arrow indicates a muscle fiber cell. Scale bar = 200 μm. (**B**) CSA of calf muscle myofibers. Quantified data are presented as a relative percentage of the control. (C) Quantification of fibers with centrally located nuclei. The data in (**B**,**C**) are presented as the means ± SD. This experiment was performed with ten mice per group. # *p* < 0.05, DEX vs. control. * *p* < 0.05, DEX + CP (0.25 or 0.5) vs. DEX; CP, collagen peptides; DEX, dexamethasone.

To confirm the preventive effect of CP against muscle wasting, we further assessed the histological features of the gastrocnemius muscles. Muscle atrophy can decrease fiber CSA, and the centralized nuclei are a symptom observed in diseased muscles. Therefore, we stained the gastrocnemius muscle with H&E, quantitatively analyzed fiber CSA, and quantified fibers with central nuclei (Figure 2A–C). As shown in Figure 2B, the CSA of the muscle was lower in the DEX group than in the control group, and it increased in the DEX+CP groups (0.25 and 0.5 g/kg) (Figure 2B). In addition, fibers with central nuclei were increased by DEX, but decreased in the DEX+CP 0.5 group (Figure 2C).

### 2.3. CP Suppresses the Myostatin/Smad Pathways Activated by DEX

**Figure 3:**
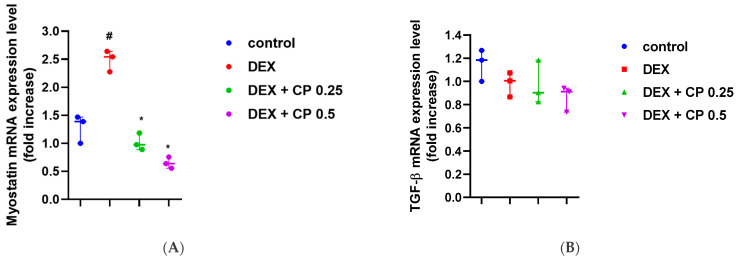

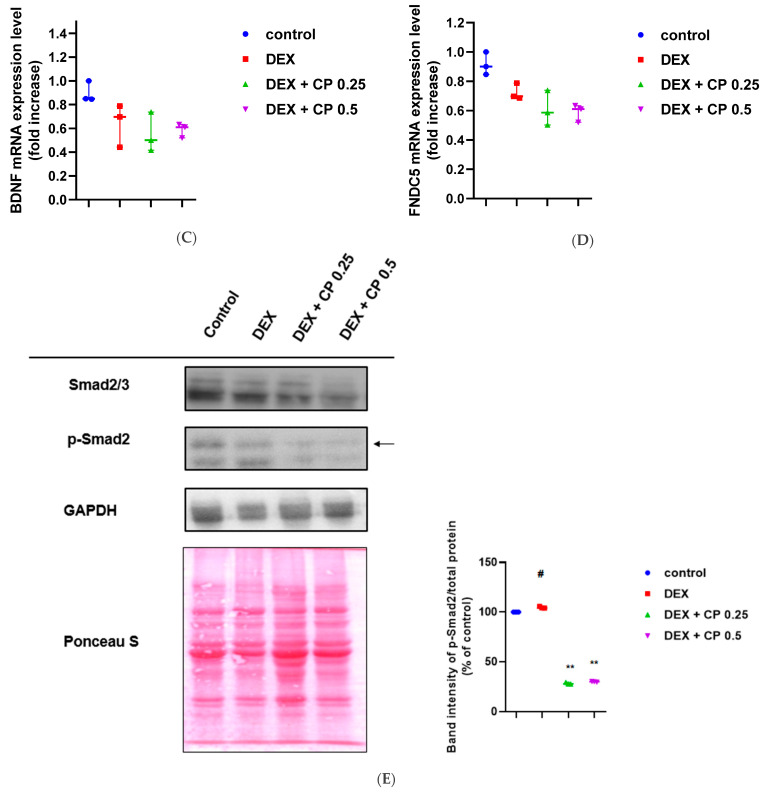

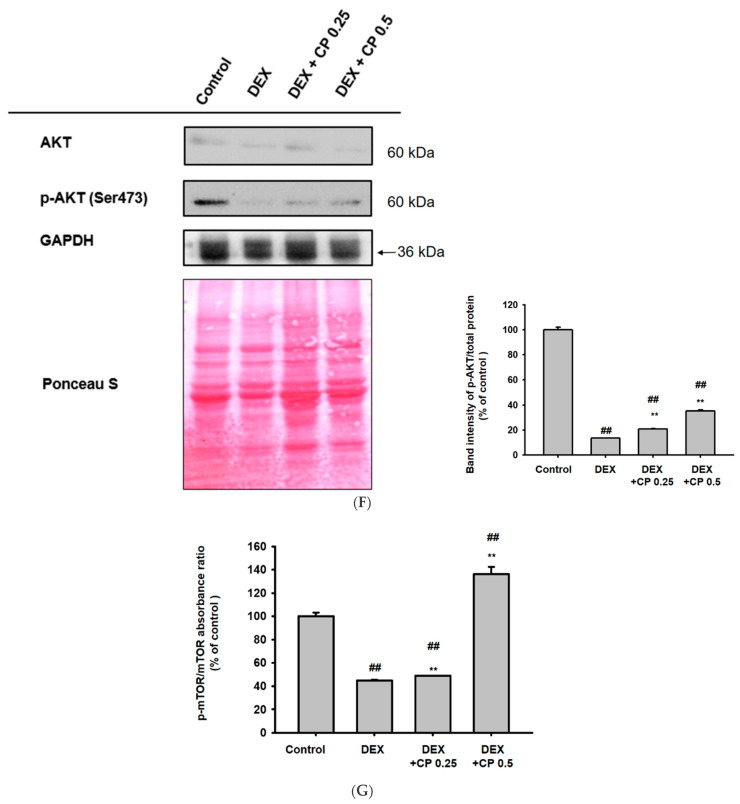
Effect of CP on myokine alterations. (**A**–**D**) The mRNA expression of myostatin (**A**), TGF-β (**B**), BDNF (**C**), and FNDC5 (**D**) in the muscles was assessed using real-time PCR. (**E**,**F**) Total Smad2/3, phospho-Smad2, total-AKT, phospho-AKT, and GAPDH levels in the muscles were assessed by immunoblotting. Total proteins were stained by Ponceau S. ImageJ was used to measure and quantify the band intensity. Arrow in (**E**) indicates p-Smad 2, and this band was used to quantify the p-Smad2 level. The p-Smad2 and p-AKT levels were normalized by Ponceau S staining. (**G**) Total and phospho-mTOR levels were analyzed by ELISA. The p-mTOR levels were normalized against the total mTOR levels. Data are presented as the means ± SD of values obtained from seven biological replicates. #*p* < 0.05, ## *p* < 0.01, DEX vs. control. # *p* < 0.05, ## *p* < 0.01, DEX + CP (0.25 and 0.5) vs. control. * *p* < 0.05, ** *p* < 0.01, DEX + CP (0.25 and 0.5) vs. DEX; CP, collagen peptides; DEX, dexamethasone.

To understand the molecular mechanisms underlying those changes, we assessed the alteration of myokine gene expression in muscle tissues. The mRNA levels of myostatin increased in the DEX group and significantly decreased in both of the CP-administration groups (Figure 3A). On the other hand, neither DEX nor CP (0.25 and 0.5 g/kg) influenced TGF-β, BDNF, or FNDC5 expression (Figure 3B–D). Myostatin is known to be involved in Smad signaling [47]; therefore, we further examined the effect of orally administered CP on Smad phosphorylation. As with the myogenin result, CP significantly reduced p-Smad2 levels compared to the DEX group (Figure 3E). Myostatin signaling has been also reported to be dependent on the AKT/mTOR pathway. In detail, myostatin exerts its inhibitory activity in muscle differentiation via suppression of AKT/mTOR [48]. Thus, we tested whether CP could regulate AKT and mTOR. DEX decreased p-AKT and p-mTOR levels in the immunoblotting analysis and ELISA, and oral CP administration (0.25 and 0.5 g/kg) increased them (Figure 3F,G). These results suggest that CP attenuates muscle loss by targeting myostatin, inhibiting Smad2 signaling, and activating the AKT/mTOR pathway.

### 2.4. CP Did Not Affect the Expression of Atrogin-1, MuRF 1, and Myogenin, Genes Related to the Catabolic Pathway

**Figure 4:**
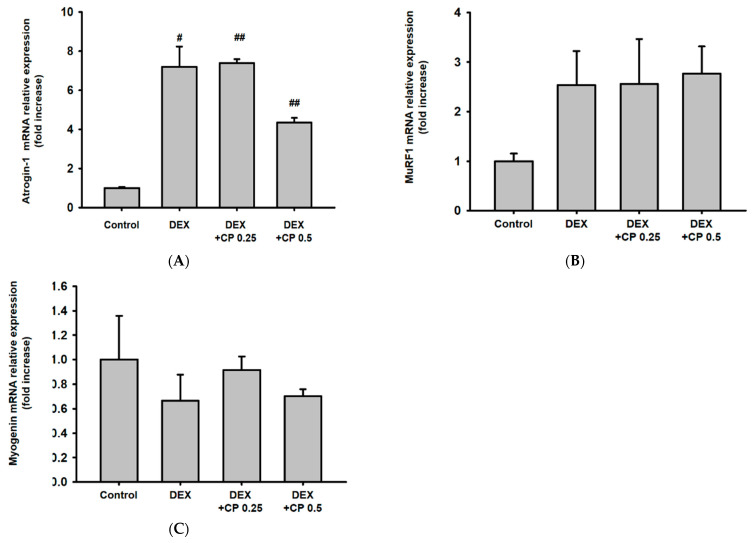
Effect of CP on muscle protein degradation pathway. (**A**–**C**) The mRNA expression of atrogin-1 (**A**), MuRF1 (**B**), and myogenin (**C**) in the muscles was assessed using real-time PCR. Data are presented as the means ± SD of values obtained from seven biological replicates. #*p* < 0.05, ## *p* < 0.01, DEX vs. control. #*p* < 0.05, ## *p* < 0.01, DEX + CP (0.25 and 0.5) vs. control. CP, collagen peptides; DEX, dexamethasone.

Because muscle protein breakdown is another essential determinant of muscle loss, we explored how CP affected degradation-related pathways. DEX tended to increase atrogin-1 mRNA expression, but CP did not affect it (Figure 4A). DEX and CP did not inhibit the expression of MuRF1 or myogenin at the transcriptional level (Figure 4B,C). These results imply that CP might prevent DEX-induced muscle atrophy in mice, regardless of the proteolysis pathway.

### 2.5. CP Decreased Serum Albumin Levels and Increased TNF-a Gene Expressions

**Figure 5:**
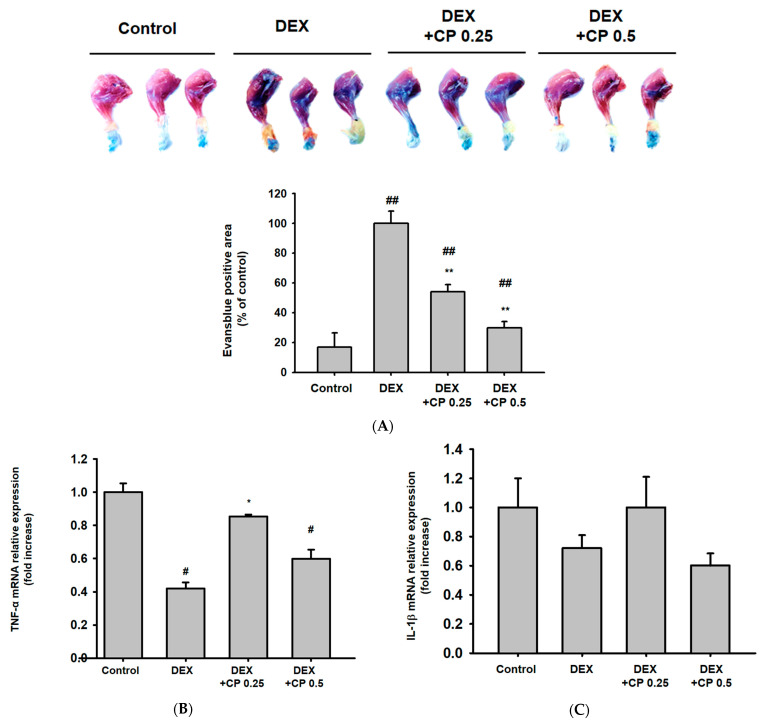

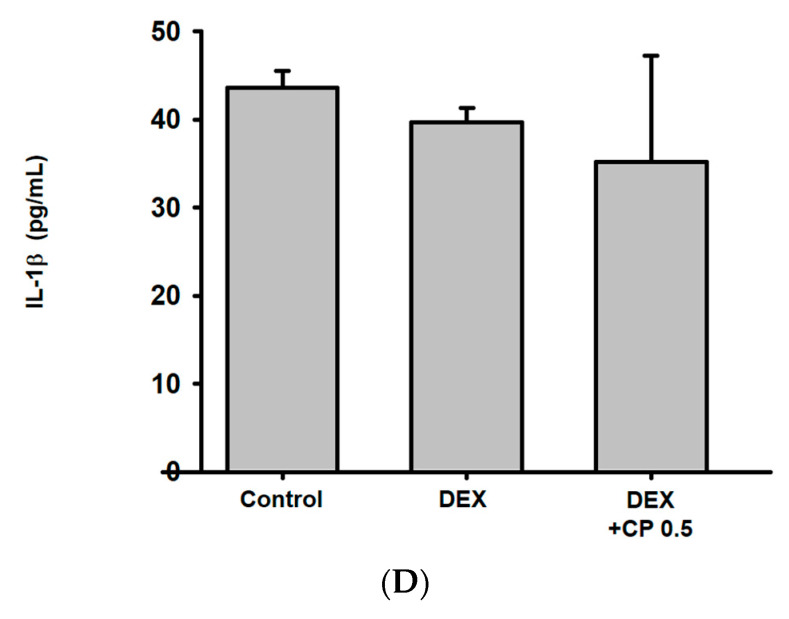
Anti-inflammatory activity of CP in muscle tissue. (**A**) Evans blue staining of the calf muscle. The blue region in the representative images indicates damaged muscle fibers. The Evans blue-positive area was quantified and is presented as a relative percentage of the control below the representative photos. This experiment was performed with three mice per group. (**B**,**C**) The mRNA expression of TNF-α and IL-1β in the muscles was assessed using real-time PCR. Data are presented as the means ± SD of values obtained from seven biological replicates. (**D**) The IL-1β levels in serum were examined using ELISA. Data are presented as the means ± SD of values obtained from three biological replicates. # *p* < 0.05, ## *p* < 0.01, DEX vs. control. # *p* < 0.05, ## *p* < 0.01, DEX + CP (0.25 and 0.5) vs. control. * *p* < 0.05, ** *p* < 0.01, DEX + CP (0.25 and 0.5) vs. DEX; CP, collagen peptides; DEX, dexamethasone.

DEX binds to serum albumin and is delivered systemically. In addition, DEX increases the biosynthesis of albumin [49]. A recent study revealed that the transport of DEX mediated by albumin binding could be a determinant of the therapeutic effectiveness of DEX [50]. Consistent with the previous studies, DEX treatment increased serum albumin levels in mice (Figure 5A). However, CP decreased the serum albumin level that was increased by DEX in a dose-dependent manner (Figure 5A). These results suggest that the decrease in serum albumin might be one of the action mechanisms of CP on DEX-induced muscle atrophy.

TNF-α is constitutively expressed in myoblasts, and increased TNF-α promotes the activation, proliferation, and differentiation of myoblasts [51,52]. Therefore, we further evaluated the effect of DEX and CP on TNF-α. Interestingly, gene expression of TNF-α was decreased by DEX, and CP (0.25 g/kg) significantly increased the reduced expression of TNF- α (Figure 5B). This result implies that induction of myogenesis by upregulation of TNF-α may be another action mechanism of CP.

Finally, DEX is extensively used as an anti-inflammatory drug, but emerging evidence has been reported that exposure to exogenous glucocorticoids induces the NLRP3 inflammasome, enhancing sensitivity to inflammatory responses [53,54]. Thus, we examined whether DEX and CP affect the expression and secretion of IL-1β, which is activated in an inflammasome-dependent manner. Gene expression of IL-1β was not affected by DEX and CP (Figure 5C). In addition, DEX and CP (0.5 g/kg) did not alter IL-1β secretion (Figure 5D). This result indicates that the preventive effect of CP on muscle atrophy is independent of the NLRP3 inflammasome.

## 3. Discussion

Skeletal muscle is a vital site for glucose and lipid homeostasis and a reservoir of amino acids. In addition, a loss of muscle mass can affect physical movement, respiration, and general metabolism throughout the body [55]. In particular, excessive muscle loss can be a poor prognostic indicator of severe diseases such as cancer, tuberculosis, and acquired immunodeficiency syndrome [56]; therefore, research on the etiology and pathologic mechanisms of and potential therapies for muscle atrophy has been extensive and ongoing. Nevertheless, no successful treatment for muscle wasting has yet been found due to its multifactorial characteristics. Recently, protein supplements, including collagen, have emerged as a treatment for muscle atrophy. Previous randomized controlled trials have demonstrated the effect of collagen on muscle mass and strength [43,44], so we focused more on understanding the molecular mechanisms of CP associated with muscle mass gain in this study.

Various techniques for targeting novel signals have been tested to block muscle protein degradation, and myostatin inhibitors have been proposed as promising candidate therapeutics [57]. Interestingly, we found here that CP modulated myostatin signals, which prevented muscle loss. These results suggest that CP might be an excellent nutritional intervention for treating muscle atrophy. Two previous methods for blocking myostatin have been tested. First, a humanized myostatin antibody (LY2495655) can directly neutralize myostatin [58,59]. Second, a soluble antibody against the myostatin receptor, ActRII, can interfere with myostatin signaling. Unfortunately, most myostatin inhibitors also block TGF-β and, thus, have adverse effects [60,61,62,63]. However, in this study, CP suppressed myostatin expression only at the transcriptional level and did not affect TGF-β. This suggests that CP might be another good option for myostatin inhibition.

In the case of irisin, several studies have reported its role in preventing muscle atrophy [18,19,20], but the most recent clinical study has shown that serum irisin levels do not affect muscle-related factors in sarcopenia in the elderly [64]. As such, the role of irisin in muscle atrophy is controversial. Because muscle atrophy has a variety of pathophysiology, including denervation, muscle disuse, and imbalance of essential amino acids or protein synthesis/degradation, inconsistent outcomes for irisin’s role may be a result of different pathological mechanisms in each type of muscle wasting. Our findings show that DEX and CP do not influence the expression of FNDC5 (a cleaved form of irisin). These results indicate that muscle atrophy induced by DEX and the preventive properties of CP in relation to muscle atrophy are independent of irisin. However, there is still insufficient evidence to correlate muscle atrophy with irisin, so further research is needed.

The precise mechanism of how myostatin regulates AKT/mTOR signaling is not fully understood. Thus far, the best-known mechanism is that the regulation of specific miRNA expression by myostatin is associated to AKT activity. Myostatin decreases the expression of microRNAs, such as miR486 and miR29, and the reduction of these miRNAs leads to the upregulation of phosphatase and tensin homolog (PTEN) protein levels. [65,66]. PTEN acts competitively with AKT against phosphatidylinositol-3,4,5 trisphosphate (PIP3) to reduce the recruitment of AKT to the membrane, thereby inhibiting AKT phosphorylation and activity [67]. Interestingly, the Smad signal has been reported to decrease miR29 and PTEN levels, causing AKT suppression [68]. Based on these reports and our findings, we would like to argue that CP targets the myostatin/Smad/AKT axis to prevent muscle atrophy. Meanwhile, the effect of CP on p-AKT/p-mTOR was weaker than that on myostatin reduction, probably because the main target of CP is myostatin signaling, and myostatin and AKT only partially interact with each other. CP was more effective at inhibiting p-mTOR than AKT. This is probably because AKT is not the only regulator of mTOR. In this study, fluctuations were observed not only in p-AKT but also in the expression of AKT. DEX showed a pattern of weakly reducing AKT expression and CP showed a pattern of restoring it, likely because DEX transcriptionally modulated AKT expression through the downregulation of AP-1 [69,70]. On the other hand, CP is expected to increase the activity of AP-1 and restore AKT expression, but further research is needed on this hypothesis. AKT/mTOR is a representative protein anabolic-stimulating signal. Thus, activating the protein synthesis pathway through mTOR/AKT could be proposed as the mechanism by which CP alleviates muscle atrophy.

Our study suggests a decrease in serum albumin levels as another action mechanism of CP. DEX acts systemically via binding to the albumin in the blood, and CP appears to block this process by reducing albumin synthesis.

**Figure 6:**
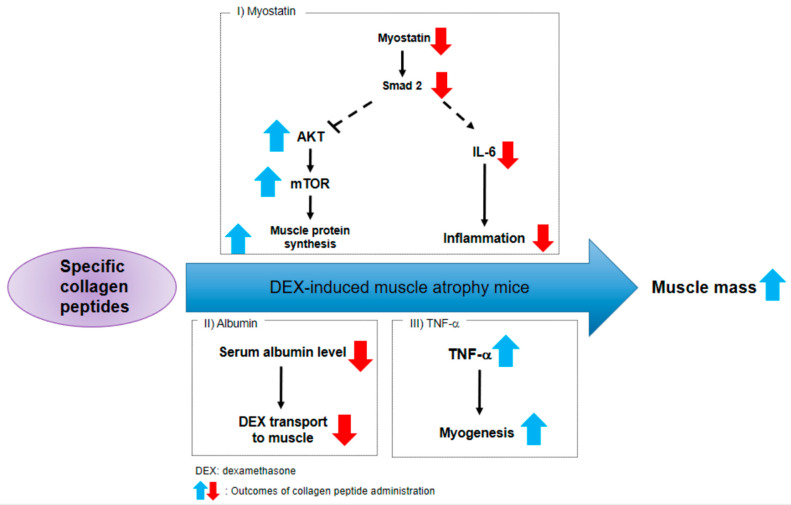
Schematic diagram presenting the mechanism for CP’s anti-muscle atrophy effects.

As depicted in Figure 6, our results strongly suggest that CP administration can ameliorate muscle atrophy by extensively suppressing myostatin signaling and activating muscle protein synthesis. Furthermore, we propose a decrease in serum albumin and an increase in TNF-α as the other mechanisms of CP action on DEX-induced muscle atrophy (Figure 6).

We employed 1 g/kg whey protein as a positive control in this study. Interestingly, the effect of collagen (0.5 g/kg) on increasing the weight of gluteus maximus and pectoralis muscles was similar to, or superior to that of whey protein (1 g/kg). It is well known that the increase in muscle synthesis by whey protein is due to the action of BCAAs. However, collagen is relatively deficient in BCAAs, so it is believed that the mechanism of action of the two proteins would be different. Collagen contains abundant glycine and arginine, and the effect of collagen may come from these amino acids. Those amino acids are known to be involved in creatine synthesis, and it has been reported that creatine can alleviate muscle wasting by modulating muscle protein catalytic and anabolic pathways [71]. In addition, glycine and arginine supplements prevent muscle atrophy via mTOR signaling [72]. Those previous reports provide a rationale for the anti-muscle atrophy effects of collagen. Indeed, our study shows that CP could decrease muscle loss by regulating myostatin expression in mice with DEX-induced muscle atrophy, which provides new insights into the potential use of CP. Based on the reports that collagen in combination with resistance exercise increased muscle strength in elderly sarcopenic men and premenopausal women [43,44], we believe that collagen intake along with exercise would be helpful to improve muscle strength. In addition, endogenous GCs can be secreted under the conditions of low nutrient availability and transmit energy stress signals that promote muscle atrophy to the organism. We, therefore, suspect that increased nutrient availability with CP intake may have conferred resistance to muscle atrophy. So far, the purpose of collagen intake has been mainly limited to improving skin [73]. However, recent randomized controlled trials and our in vivo studies suggest that CP can be applied to prevent muscle atrophy [43,44,45]. Even if rodents and humans have the same myostatin gene, the expression pattern or way to regulate its expression may be different. This likely means that there is no guarantee for us to observe the same activity seen in mice in human clinical trials. Therefore, further investigation into the effect of CP intake on myostatin levels in humans is necessary.

The mode of absorption of CP is expected to be similar to that of dietary protein. Dietary proteins are generally degraded by digestive enzymes and then absorbed in the form of dipeptides or tripeptides. Similarly, peptide quantification by mass spectrometry demonstrated that di- and tripeptides are present in circulating plasma after oral administration of collagen hydrate [74]. Even though absorbed peptides derived from collagen can be distributed to various tissues, including muscle [75], the transport mechanisms to muscle cells have not been clearly elucidated. Because the cell permeability, bioavailability, and tissue distribution of collagen depend on the collagen source, type, and size, further experiments are needed to accurately understand the absorption and bioavailability kinetics of CP. Moreover, collagen is a safe, non-toxic dietary supplement, and collagen-derived peptides, such as Gly-Pro-Hyp and Pro-Hyp, are known to be stable in gastrointestinal fluid and plasma for 2 h in rats [76]; therefore, we believe that CP would be stable in vivo. At least 29 di- and tripeptides have been identified in plasma after oral administration of collagen, and these di- and tripeptides are reported to exhibit various biological activity. For example, prolyl hydroxyproline, a representative dipeptide that is derived from collagen, is known to directly bind to Foxg1 and regulate the expression of certain genes [77]. Similarly, in myostatin regulation, it is estimated that di- or tripeptides from collagen digestion act as signal molecules and specifically regulate the expression of myostatin by binding to a specific transcriptional factor. However, further studies are needed for a clear understanding of the regulatory mechanisms of myostatin expression by CP.

## 4. Materials and Methods

### 4.1. Materials and Reagents

CP, which is BODYBALANCEI^®®^ P from GELITA AG, was provided by COSMAX NS (Seongnam, Korea). The amino acid composition of CP is presented in Table S1 (see Supplementary Material), as similarly reported previously [43]. The average molecular weight of CP is 3 kDa. TRIzol reagent was manufactured by MRC (Cincinnati, OH, USA). For the polymerase chain reaction (PCR) analyses, Probe Blue Mix Hi-ROX was purchased from PCR Biosystems (Archway Road, London, UK). DEX and sodium dodecyl sulfate were purchased from Sigma Chemical Co. (St. Louis, MO, USA). Ethanol, methanol, and isopropanol were obtained from DAEJUNG (Seoul, Republic of Korea). Antibodies specific for both the total forms and phosphorylated forms were acquired from Cell Signaling Technology (CST, Danvers, MA, USA) and Santa Cruz Biotechnology (Dallas, TX, USA).

### 4.2. Animal Experiment

The 5-week-old male ICR mice were provided by Orient Bio (Seongnam, Korea) and housed for a week for acclimation. After acclimation, 40 mice were randomly classed to the control, DEX, DEX+CP 0.25 (g/kg), and DEX+CP 0.5 (g/kg) groups. To induce DEX-mediated muscle atrophy, 0.01% (*w*/*v*) DEX in drinking water was given to the DEX, DEX+ CP 0.25, and DEX+ CP 0.5 groups daily. DEX in drinking water was provided daily and mice had free access to DEX in water, as reported previously [78,79,80]. The control group received only tap water. CP powder was dissolved in a 0.5% carboxymethyl cellulose (CMC) solution and orally administered by gavage daily. A 0.5% CMC solution without CP was orally administered to the control and DEX groups. We provided restricted food to exclude the possibility of muscle increase or decrease due to feed intake variation between groups and to assess only the effect of the CP supplementation. All mice were given 15% of their body weight food daily, which is the appropriate daily intake. On two days of every week, all of the mice were exercised on a treadmill for 40 min, as indicated in Figure 1A. The experiment was conducted for 4 weeks, and then the mice were sacrificed with isoflurane. Animal care was based on guidelines issued by the National Institutes of Health for the Care and Use of Laboratory Animals (NIH Publication 80-23, revised in 1996). This study was conducted according to guidelines established by the Institutional Animal Care and Use Committee (IACUC) of Sungkyunkwan University (SKKUIACUC2021-05-44-1).

### 4.3. Micro-Computed Tomography (microCT)

After sacrifice, each mouse’s gastrocnemius muscle was dissected and fixed with 4% formalin solution for 2 days. The solution for each sample was changed to phosphate buffered saline (PBS) to remove leachate 24 h before the microCT. The gastrocnemius muscles were scanned 360° around in vertical rotation steps of 0.6° with a Skyscan 1076 MicroCT scanner (Skyscan, Kontich, Belgium) at a 5 mm resolution. Using CTAn software (version 1.18), regions of interest (ROIs) were analyzed based on cross-sectional images, and volumes of interest in each sample were calculated by summing the ROIs. MicroCT scanning was conducted at the Center for University-Wide Research Facilities at Jeonbuk National University.

### 4.4. Muscle Histology

Paraffin-blocked gastrocnemius tissue samples were sliced to 4 μm thicknesses on slide glass. After we dipped the samples into xylene to eliminate the paraffin embedding, we serially put them put into 100%, 95%, and 70% ethanol. The samples were then stained with eosin (Abcam, ab246824) for 3 min and rinsed in PBS 3 times. After the samples had been washed, they were dehydrated with 70%, 95%, and 100% ethanol, and cover glasses were mounted over the stained sections. The sections were then examined under a microscope (Eclipse TE 2000-U, Nikon, Dusseldorf, Germany), and the cross-sectional areas (CSAs) were quantified using ImageJ software. A total of 3 slides per group were analyzed and 60 myofibers per muscle were quantified.

Muscle fibers with central nuclei were quantified following hematoxylin and eosin staining and were presented as a % of central fibers to total fibers.

### 4.5. RNA Extraction and cDNA Synthesis

TRIzol reagent and 1-bromo-3-chloropropane were used to isolate RNA from muscle tissue. Then, complementary DNA (20 μL) was synthesized from 1000 ng of RNA using a Thermo Fisher cDNA synthesis kit (Waltham, MA, USA).

### 4.6. Real-Time PCR

Real-time PCR was conducted using qPCRBIO SyGreen Blue Mix Lo-ROX (PCR Biosystems Ltd., London, UK) according to the manufacturer’s instructions. The PCR reaction was performed using a real-time CFX96 thermal cycler (Bio-Rad Laboratories Inc., Hercules, CA, USA) under the following conditions for 44 cycles: 10 s denaturation time at 95 °C, 10 s annealing time at 58 °C, and 60 s extension time at 72 °C. The expression of the atrogin-1, MuRF1, myogenin, myostatin, FNDC5, BDNF, TGF-β, and IL-6 genes was normalized to GAPDH and is expressed as the fold increase. The sequences of the primers used in this study are given in Table 1.

### 4.7. Immunoblotting

Sectioned muscles were lysed with ice-cold RIPA buffer (20 mM Tris-HCl, pH 7.4; 2 mM EDTA; 2 mM ethylene glycol tetraacetic acid; 1 mM DTT; 50 mM β-glycerol phosphate; 0.1 mM sodium vanadate; 1.6 mM pervanadate; 1% Triton X-100; 10% glycerol; 10 μg/mL aprotinin; 10 μg/mL pepstatin; 1 μM benzamide; and 2 μM PMSF). After homogenization, the protein lysates were centrifuged at 12,000 rpm for 5 min at 4 °C, and only the supernatants were used. To quantify the total proteins in the tissue lysates, a Bradford assay was conducted. A total of 20 μg/μL of protein was loaded onto each 10% polyacrylamide gel. Those gels were electrophoresed at 100 V for 2 h. The proteins were sorted by size and transferred to a PVDF membrane overnight. That membrane was probed with the following primary antibodies overnight at 4 °C: anti-Smad2/3 (CST #8685), anti-p-Smad2 s245/250/255 (CST #3104), anti-p-Smad2 s465/467/Smad3 (CST #8828), anti-AKT (CST #9272), anti-p-AKT Ser473 (CST #4058), and anti-GAPDH (Santa Cruz #SC166545). The membrane was washed 3 times with Tris-buffered saline with Tween 20 and then incubated with secondary antibodies conjugated with horseradish peroxidase in BSA for 2 h. Chemiluminescence was detected with a ChemiDoc imaging system (Luminograph III, ATTO, NY, USA), and band intensity was measured and quantified with ImageJ software.

### 4.8. mTOR ELISA

Sectioned muscles were lysed with RIPA buffer. To quantify the activity of mTOR and p-mTOR, Pathscan sandwich ELISA kits (mTOR: CST#7974, p-mTOR: CST#7976) were used. A total of 100 μL of muscle lysates were duplicated for loading, and ELISA was conducted by following the manufacturer’s guidelines. Absorbance was measured at 450 nm.

### 4.9. Evans Blue Staining

On day 27, right after the treadmill exercise, the mice were intraperitoneally injected with tribromoethanol to anesthetize them. Evans blue dye was injected at the tail vein, and then the mice were sacrificed, and their legs were dissected with a scalpel and rinsed in room temperature PBS. Then, we put the rinsed tissue into formamide for 72 h at room temperature to infuse the Evans blue. A total of 50 μL of each Evans blue-infused sample was placed on a 96-well plate, and their absorbance was detected at 620 nm.

### 4.10. Statistical Analysis

Animal experiments were performed with 10 mice per group. Data in the figures are expressed as means ± standard deviations (SDs) and calculated with GraphPad Prism software (Ver 8.0). All data were also transformed into scatterplots which contain median and sample size, as indicated by Weissgerbers et al. [81]. One-way analysis of variance (ANOVA) was used in Figure 1G, followed by a Dunnett’s post hoc test. Data in Figure 1D–F, Figure 2B, Figure 3A–G, Figure 4A–C and Figure 5A–D were statistically analyzed by Student’s t-test. For all analyses, *p* < 0.05 was considered to be statistically significant.

## 5. Conclusions

In conclusion, we investigated the effects of specific a CP on muscle atrophy and examined its molecular mechanisms in mice with DEX-induced muscle atrophy by orally administering CP for four weeks. As shown in the microCT analysis, CP (0.5 g/kg) intake significantly increased calf muscle volume in mice with DEX-induced muscle atrophy, restored the weight of the gluteus maximus and pectoralis major, and increased the fiber CSA. CP significantly inhibited the mRNA expression of myostatin and the phosphorylation of Smad2, but it did not affect the expression of the TGF-β, BDNF, or FNDC5 genes. In addition, AKT/mTOR, a central pathway for muscle protein synthesis, was enhanced in the groups that were administered CP, as summarized in Figure 6. These data strongly indicate that CP can alleviate muscle wasting through a multitude of mechanisms and could potentially be used as a supplement or treatment to prevent muscle atrophy.

## Figures and Tables

**Table 1:** Primer sequences for mouse genes.

Gene	Direction	Primer sequence (5′→3′)
BDNF	F	TACCTGGATGCCGCAAACAT
	R	TGCTTCAGTTGGCCTTTGGA
FNDC5	F	CACGCGAGGCTGAAAAGATG
	R	GAGCTATAACACCTGCCCACA
Myostatin	F	TCACGCTACCACGGAAACAA
	R	AGGAGTCTTGACGGGTCTGA
Myogenin	F	AGGAGATCATTTGCTCGCGG
	R	GTTGGGCATGGTTTCGTCTG
TGF-β	F	AACAATTCCTGGCGTTACCTT
	R	CTGCCGTACAACTCCAGTGA
IL-6	F	AGCCAGAGTCCTTCAGAGAGAT
	R	AGGAGAGCATTGGAAATTGGGG
MuRF1	F	GAGGGGCTACCTTCCTCTCA
	R	AGAGGAACGCTGCCTTTCAA
Atrogin-1	F	TTCAGCAGCCTGAACTACGA
	R	AGTATCCATGGCGCTCCTTC
GAPDH	F	GGTTGTCTCCTGCGACTTCA
	R	CATTGAGAGCAATGCCAGCC

## Data Availability

The data are contained within the article.

## Supplementary Materials

The following are available online at https://www.mdpi.com/article/10.3390/molecules28041950/s1, Table S1: Amino acid composition of the collagen peptides.

## Sample Availability

Samples of the compounds are available from the authors.
