# Supplementary material for: The Preventive Effect of Specific Collagen Peptides against Dexamethasone-Induced Muscle Atrophy in Mice
Source: Molecules. 2023 Feb 18;28(4):1950. doi: 10.3390/molecules28041950 (PMC9960993; doi:10.3390/molecules28041950)
Supplement: Supplementary file 1 [file molecules-28-01950-s001.zip › molecules-2138017-supplementary.pdf]

Supplementary table S1. Amino acid composition of CP.

**Amino acid**	**Weight (%)***
Glycine	22.1
Proline	12.3
Alanine	8.5
Hydroxylproline	11.3
Glutamic acid	10.1
Arginine	7.8
Aspartic acid	5.8
Serine	3.2
Lysine	3.8
Leucine	2.7
Valine	2.4
Threonine	1.8
Phenylalanine	2.1
Hydroxylysine	1.7
Isoleucine	1.3
Methionine	0.9
Histidine	1.2
Tyrosine	0.9

* weight % of each amino acid in CP.
